# Thirty years of public speaking anxiety research: topic modeling and semantic trend forecasting using LDA–Word2Vec integration

**DOI:** 10.3389/fnhum.2025.1653899

**Published:** 2025-10-24

**Authors:** Mocheng Lin, Yi Sun, Lang Chen

**Affiliations:** ^1^School of Foreign Studies, Fuzhou University of International Studies and Trade, Fuzhou, China; ^2^Foreign Linguistics and Applied Linguistics Research Center, Guangdong University of Foreign Studies, Guangzhou, China

**Keywords:** public speaking, bibliometric analysis, topic evolution, hotspot prediction, LDA, Word2Vec

## Abstract

Public speaking anxiety (PSA) is a widespread condition with profound psychological, educational, and occupational consequences. Despite increasing academic attention, few studies have systematically mapped the thematic development of PSA research or anticipated future directions. This study employs a semantically enriched framework integrating latent Dirichlet allocation (LDA) with Word2Vec to analyze 990 peer-reviewed journal articles (1995–2024) retrieved from the Web of Science Core Collection, spanning SSCI, SCI-Expanded, ESCI, and A&HCI indices. Nine major thematic clusters are identified, covering topics such as mental health distress, physiological reactivity, language apprehension, social phobia, and virtual reality interventions. Thematic evolution across four historical phases reveals growing conceptual convergence and an increasing focus on digital anxiety and assessment technologies. A Sankey diagram illustrates patterns of thematic continuity and transformation, highlighting both enduring topics and emerging lines of inquiry. To predict future research frontiers, two novelty indicators—Cluster Novelty and Topic Novelty—are introduced. Clusters focused on language learning (Topic 4), virtual therapy (Topic 6), and psychometric tools (Topic 8) emerge as the most temporally novel. This study provides a comprehensive, data-driven mapping of PSA research and offers methodological guidance for future interdisciplinary bibliometric analyses.

## 1 Introduction

### 1.1 Research on public speaking anxiety

Public speaking is a foundational communicative competence essential in both educational and professional settings. Within educational contexts, public speaking serves as a primary method for assessing students’ knowledge and comprehension, and is acknowledged as a critical component for fostering graduates’ employability (Caballero and Walker, 2010; Joughin, 2007). Professionally, proficiency in public speaking often surpasses the perceived value of traditional technical skills, underscoring its critical role in career success (Blume et al., 2013; Robles, 2012). Recent studies also highlight its increasing importance in digital and cross-cultural environments, where effective public speaking must adapt to diverse audiences, virtual platforms, and multilingual contexts (Butler, 2017; Zmait and Rasheed, 2025).

Despite its recognized importance, public speaking commonly evokes anxiety, worry, apprehension, and, in severe cases, panic and avoidance behaviors. Public speaking anxiety—often referred to as glossophobia (Bodie, 2010)—is interchangeably referred to as fear of public speaking (FoPS; Blöte et al., 2009) and speech anxiety (Mandeville, 1991), highlighting its dual psychological and physiological dimensions. Approximately 63% of the general population report experiencing some level of public speaking anxiety (Marinho et al., 2017), with around 61% of college students reporting it as their most common fear (Dwyer and Davidson, 2012). Typically emerging during adolescence, PSA is frequently identified as a specific form of social anxiety disorder (Blöte et al., 2009), significantly impairing up to 97% of individuals who experience social anxiety (Beidel and Turner, 1998). Consistently recognized as one of the most commonly feared situations among university students and community populations alike (Pollard and Henderson, 1988; Holt et al., 1992; Tillfors and Furmark, 2007), PSA substantially impacts individuals’ quality of life, academic performance, occupational success, and social relationships (Fehm et al., 2005; Stein and Kean, 2000). In addition, research has shown that PSA can lead to maladaptive tendencies, such as negative cognitive biases, avoidance, poor speech preparation, and dropout, resulting in missed educational, social, and professional opportunities (Daly and McCroskey, 1984; Bodie, 2010). These negative effects are also linked to reduced self-efficacy, lower interpersonal motivation, and heightened physiological stress responses during speech performance (Paradewari, 2017; Beatty and Behnke, 1991; Gonzalez-Bono et al., 2002). If untreated during adolescence and adulthood, PSA may progress into generalized SAD, further intensifying its disabling consequences (Gregory et al., 2007; Hofmann et al., 1999).

Technological interventions, such as virtual reality (VR) exposure therapy and structured online speaking exercises, have shown promising results in reducing PSA and improving speaker confidence (North et al., 1998; Harris et al., 2002; Broeckelman-Post et al., 2019). Such developments underscore the interdisciplinary nature of PSA research, which increasingly integrates psychological, educational, technological, and cross-cultural perspectives. Given the high prevalence, early onset, and substantial negative outcomes associated with PSA, it remains a critical interdisciplinary research topic, intersecting the fields of psychology, education, and communication.

### 1.2 Topic models and word embeddings

In response to the expanding volume and diversity of publications, text mining and natural language processing (NLP) have become essential parts of bibliometric research. Among various techniques, latent Dirichlet allocation (LDA) has emerged as one of the most widely adopted topic modeling methods, owing to its probabilistic framework and relative interpretability (Blei et al., 2003). By treating documents as mixtures of latent topics and topics as distributions over words, LDA enables researchers to uncover hidden thematic structures within large collections of academic articles. This automated process reduces human bias and provides insights into how different topics emerge, converge, or decline across specified time frames (Huang et al., 2021).

While LDA excels at revealing the global contours of a research field, it relies heavily on word co-occurrence patterns and can overlook subtler semantic relationships between terms, especially those that appear with lower frequency. To address this limitation, word embeddings such as Word2Vec have been incorporated into bibliometric analyses to capture deeper semantic and syntactic relationships (Mikolov et al., 2013). Word2Vec trains dense vector representations of words based on their surrounding context, enabling the identification of terms that are semantically related even if they do not frequently co-occur in the corpus. When applied in tandem with LDA, word embeddings can enhance topic interpretability, improve the detection of emerging concepts, and reveal latent connections between subfields (Shi et al., 2017; Li et al., 2018).

The complementarity between LDA and Word2Vec has been demonstrated in several domains. For example, Ma et al. (2023) integrated LDA and Word2Vec to track the evolution of mental models from global to local perspectives; Zhang et al. (2023) applied a similar hybrid method to examine cross-domain research collaboration; Kim et al. (2020) proposed a W2V–LSA approach for blockchain technology trend analysis; and Al Tawil et al. (2024) incorporated semantic embeddings into phishing email detection to enhance classification performance. Building on these methodological foundations, the present study adapts the LDA–Word2Vec framework to the interdisciplinary domain of public speaking anxiety, which spans psychology, education, and communication. In doing so, we tailor the integration pipeline to track thematic evolution across distinct developmental phases and forecast future research hotspots, thereby leveraging the strengths of prior studies while addressing the unique thematic dynamics of PSA research. Such a hybrid methodological framework is particularly apt for exploring multifaceted, interdisciplinary fields like public speaking, where cross-pollination with psychology, education, leadership, and digital communication leads to rapidly evolving research frontiers.

Compared to alternative topic modeling approaches—such as non-negative matrix factorization (NMF), latent semantic analysis (LSA), Top2Vec, and BERTopic—our choice of LDA combined with Word2Vec offers several advantages. NMF and LSA, while effective in dimensionality reduction, often lack the probabilistic interpretability of LDA. Top2Vec and BERTopic leverage embeddings directly and can automatically determine topic numbers, but their performance is sensitive to embedding quality and clustering hyper parameters, and they may produce less interpretable topic-word distributions in highly interdisciplinary corpora. In contrast, the integrated LDA–Word2Vec framework used in this study retains the transparency and theoretical grounding of probabilistic topic modeling while benefiting from Word2Vec’s ability to capture semantic similarity, making it especially suitable for the multifaceted and interdisciplinary nature of public speaking research.

### 1.3 Research significance and problem statement

Previous reviews and meta-analyses of PSA have primarily emphasized psychological treatments, predictive factors, and coping mechanisms. Ebrahimi et al. (2019), for example, highlighted promising psychological interventions, advocating for further longitudinal research. Similarly, research by Lestari et al. (2021) identified significant predictors, such as self-efficacy and audience interactions, noting their inverse correlation with anxiety severity. Additionally, coping strategies among university students, especially concerning language proficiency and emotional management, have received focused attention (Tee et al., 2020).

Despite these valuable insights, existing reviews often rely heavily on qualitative descriptions or narrow quantitative assessments, lacking comprehensive, data-driven analyses of long-term thematic evolution. Thus, current scholarship inadequately represents how public speaking anxiety research topics have shifted over time and fails to systematically identify emerging trends and future research directions.

To address this gap, this study employs a novel integrated approach combining LDA and Word2Vec to provide a semantically enriched, longitudinal analysis of public speaking anxiety research from 1995 to 2024. Specifically, this study aims to address three primary questions:

1. What are the dominant research topics in the field of public speaking anxiety, as revealed through LDA topic modeling?
2. How have these topics evolved over time, and what thematic continuities or transformations can be observed across distinct historical periods?
3. Which topics and clusters exhibit the highest levels of novelty in the most recent period (2020–2024), and what do these indicate about emerging research frontiers in PSA?

By addressing these questions, the current study provides a comprehensive thematic mapping of PSA literature, highlights significant thematic trajectories, and predicts potential research hotspots. The methodological integration further enriches interpretability and can inform future interdisciplinary investigations and practical interventions across educational, psychological, and communication contexts.

## 2 Methodology

**Figure 1:**
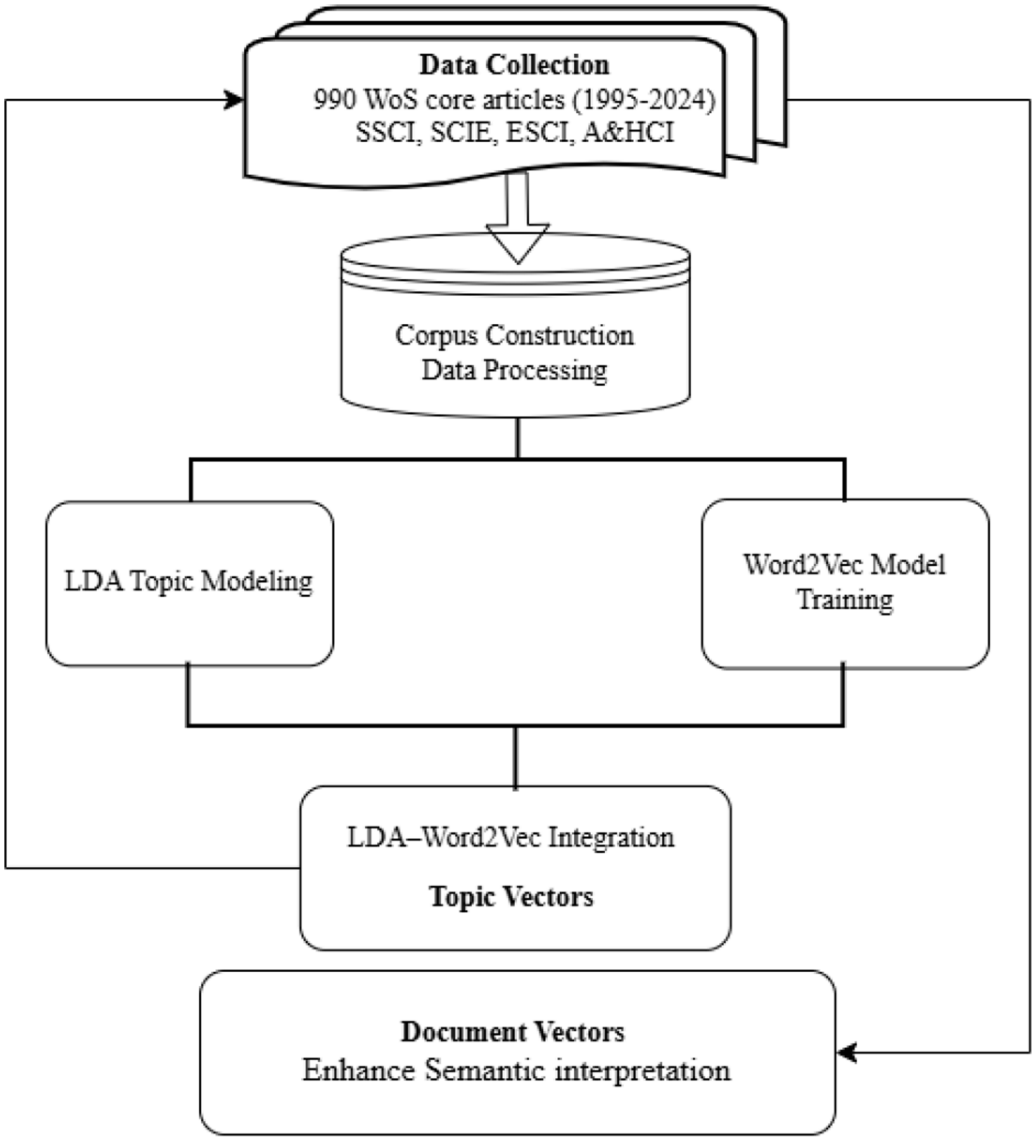
Methodological workflow of the study.

As illustrated in Figure 1, this study adopts a multi-stage analytical framework to examine the thematic evolution of public speaking anxiety (PSA) research from 1995 to 2024. The analytical process includes data collection, preprocessing, modeling through latent Dirichlet allocation (LDA) and Word2Vec, integration of these models, and semantically enriched analysis to identify thematic trends and forecast emerging research hotspots.

### 2.1 Data collection

This study explores the thematic development of public speaking anxiety (PSA) research over a 30-year period by constructing a targeted corpus of peer-reviewed journal articles retrieved from the Web of Science Core Collection. A comprehensive search was conducted using topic-level keywords such as “public speaking anxiety,” “speech anxiety,” “fear of public speaking,” and “glossophobia.” The search was limited to articles written in English and classified as the document type “Article.” The time span was set from January 1, 1995 to December 31, 2024, providing a longitudinal view of research progression.

To maintain academic rigor and disciplinary diversity, the search results were restricted to articles indexed by the Social Sciences Citation Index (SSCI), Science Citation Index Expanded (SCI-Expanded), Emerging Sources Citation Index (ESCI), and Arts & Humanities Citation Index (A&HCI). Following initial retrieval, 11 articles lacking abstracts were removed to ensure suitability for semantic analysis, resulting in a refined dataset of 990 articles.

**Figure 2:**
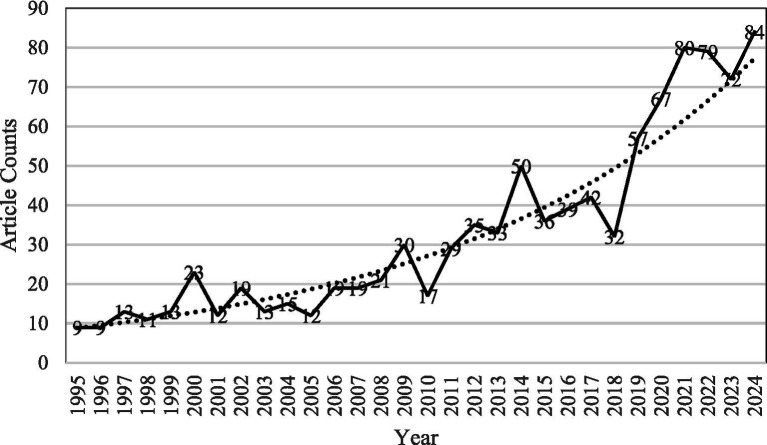
Annual publication trends in public speaking anxiety research (1995–2024).

The temporal distribution of publications reveals a clear growth trajectory in PSA research (Figure 2). Based on annual publication counts, the 30-year timespan was divided into four distinct developmental phases:

- Emergent phase (1995–2004): Characterized by low and inconsistent output, this period marks the formative stage of PSA scholarship, with annual publication numbers ranging from 9 to 23.
- Early expansion phase (2005–2013): Marked by steady growth, this phase saw increased academic engagement with PSA, particularly in clinical and educational contexts.
- Consolidation phase (2014–2019): A period of accelerated growth and thematic diversification, reflecting broader interdisciplinary integration.
- High productivity phase (2020–2024): The most prolific stage to date, with annual outputs exceeding 70 articles, driven by rising interest in virtual communication, emotional regulation, and anxiety in online educational settings.

This refined and chronologically segmented corpus provides a robust foundation for subsequent LDA topic modeling, temporal trend analysis, and future research forecasting.

### 2.2 Data preprocessing and Word2Vec model training

This phase involved preprocessing the collected textual data to ensure semantic accuracy and reliability, followed by training a Word2Vec model to capture nuanced semantic relationships among words within the public speaking corpus. Initially, the corpus—which comprised article titles, abstracts, and publication years—was systematically prepared through a series of cleaning steps. Specifically, the titles and abstracts of each article were combined into a unified text document, converted to lowercase to maintain consistency, and subsequently tokenized into individual words. Non-alphanumeric characters and irrelevant tokens were removed, and a customized stop word list was employed to filter out common and uninformative words. The resulting cleaned corpus consisted of tokenized word lists for each document, which were then saved externally to enhance transparency and reproducibility.

Subsequently, the Word2Vec algorithm was applied to the cleaned corpus to generate dense, low-dimensional vector representations of words, thereby capturing their contextual semantic relationships. To identify the optimal configuration for the Word2Vec model, we systematically experimented with multiple hyper parameter settings, including various embedding dimensions (vector sizes) and context window lengths. Model performance was quantitatively assessed through coherence evaluation, specifically by calculating the average cosine similarity across predefined word pairs that exemplify meaningful semantic relationships within the corpus. Following this evaluation process, the optimal parameters were manually selected based on model coherence scores. Finally, the Word2Vec model was retrained using the identified optimal parameters, and both the complete model and its corresponding word vectors were saved to disk. This ensured that the semantic insights captured by the model could be readily utilized in subsequent analyses, including topic modeling and dynamic thematic exploration within public speaking research.

### 2.3 LDA model training, evaluation, and topic extraction

In this stage, we employed latent Dirichlet allocation (LDA) topic modeling to uncover latent thematic structures embedded within the public speaking corpus. To prepare for the LDA analysis, a dictionary was first created from the previously cleaned corpus, after which infrequent words appearing fewer than three times and overly frequent words appearing in more than 50% of the documents were systematically removed. This step ensured a concise and semantically meaningful vocabulary, balancing specificity with generalizability. Subsequently, each document was represented as a bag-of-words (BoW) vector based on this refined dictionary, providing structured input for LDA model training.

To identify the most appropriate number of topics, we systematically experimented with a range of topic numbers (from 2 to 19) while maintaining consistency in other model parameters. Each model configuration underwent rigorous evaluation using three primary metrics: (1) Coherence Score, which quantitatively assessed the semantic clarity and interpretability of the extracted topics (with higher scores indicating better semantic coherence); (2) Log Perplexity, which measured the model’s overall fit to the corpus, with values closer to zero (less negative) indicating superior model performance; and (3) Topic Overlap, a metric designed to evaluate thematic distinctiveness by quantifying the extent of shared high-frequency words between topics, with lower overlap values indicating more clearly delineated themes.

The optimal topic count was manually selected based on an integrated evaluation of these metrics. Following this determination, the final LDA model was retrained using the optimal number of topics, and the resultant model, dictionary, and BoW corpus were saved externally to ensure reproducibility. Lastly, the top words and associated probabilities for each extracted topic were examined manually to verify thematic representativeness and interpretability within the public speaking literature. This rigorous methodological approach established a solid foundation for subsequent analyses, including tracking dynamic thematic evolution and predicting future research hotspots.

### 2.4 LDA–Word2Vec integration

To enhance the semantic depth and interpretability of topic modeling results, this study implemented a fusion approach that integrates the probabilistic outputs of the LDA model with the contextual word representations generated by the Word2Vec algorithm. This integration enables a more nuanced understanding of topics and documents by combining the statistical structure of topic modeling with the semantic richness of word embeddings.

The process began by training a Word2Vec model on the cleaned corpus, which produced dense vector representations for each word based on its surrounding context. These vectors reflect how words are semantically related to one another across the entire dataset.

Next, for each topic identified by the LDA model, we extracted a set of high-probability keywords—typically the top 10 words most strongly associated with that topic. Since not all keywords contribute equally to a topic’s meaning, we assigned greater weight to words with higher probability scores. We then combined the Word2Vec vectors of these keywords, weighted according to their importance, to construct a single vector that semantically represents the entire topic. In this way, each topic was transformed from a probability distribution over words into a compact and semantically informed topic vector.

We then turned to the documents. Each document in the corpus had an LDA-generated topic distribution, showing how strongly it related to each topic. For each document, we selected the top five dominant topics—those with the highest proportions—and calculated a weighted average of their corresponding topic vectors. This resulted in a single vector representation for each document that captures both the dominant themes (from LDA) and the nuanced word-level semantics (from Word2Vec).

This integrated vector representation allowed us to compare the original 990 articles collected in the data collection process based on their thematic and semantic similarities, facilitating more meaningful clustering, visualization, and time-series analysis. It also enabled a smoother transition into downstream tasks, such as measuring topic evolution or identifying emerging areas of research, by leveraging both statistical and semantic information.

### 2.5 Semantically informed temporal clustering and evolution visualization

To trace the temporal evolution of research themes within the field of public speaking anxiety (PSA), this study employed a semantically enriched clustering approach based on the integration of LDA topic distributions and Word2Vec-based document embeddings. The fused document vectors—generated as detailed in Section 2.4—served as the input for clustering and longitudinal analysis.

First, the dataset was segmented into four time phases as defined in Section 2.1. For each phase, the semantic document vectors were standardized using *z*-score normalization and subjected to *k*-means clustering. To determine the optimal number of clusters (*k*) for each phase, we computed silhouette scores across a range of cluster values (from *k* = 2 to *k* = 10), selecting the k that maximized the average silhouette score. These optimized clusters represent thematically coherent groups of PSA-related publications within each period.

To interpret each cluster, we identified its representative document—defined as the article whose semantic vector was closest to the cluster centroid in Euclidean space. For each representative document, the dominant topic was retrieved by selecting the LDA topic with the highest probability score. To further enhance thematic interpretability, the keywords for each cluster were generated using a topic-weighted approach. Specifically, for every document, the word distributions from all LDA topics were aggregated according to their associated probabilities. The top-ranking words from this weighted set were then used as representative keywords, capturing both the dominant and secondary semantic signals.

After identifying clusters across the four time phases, we computed inter-cluster similarities using cosine similarity between the centroids of cluster vectors from adjacent time periods. Only those cluster pairs with a similarity score above a threshold (0.8) were retained, reflecting meaningful thematic continuity. These transitions were visualized using a Sankey diagram, where each node represented a semantically cohesive cluster, and each link denoted inter-temporal semantic inheritance. Additionally, the dominant LDA topic for each cluster was color-coded and attached to both nodes and links, enhancing interpretability and enabling an intuitive view of topic flows across time.

To support analytical reproducibility and enable further downstream investigation, all intermediate data—including cluster-topic matrices, representative documents, topic keyword summaries, and Sankey link structures—were saved as structured CSV files. This methodological stage provides a high-resolution view of the thematic evolution of PSA research, allowing for both qualitative interpretation and quantitative trend detection.

## 3 Results

### 3.1 Word2Vec results

A systematic evaluation of Word2Vec model parameters was conducted, experimenting with vector sizes (50, 100, 200, and 300) and context window sizes (2, 5, and 10). Semantic coherence scores were computed based on the average cosine similarity across predefined domain-specific word pairs related to public speaking anxiety. The optimal Word2Vec configuration was determined to be a vector size of 200 and a context window of 2, achieving the highest coherence score of 0.7570 (Table 1), indicating superior capturing of academic linguistic patterns within the corpus.

**Table 1:** Parameter evaluation for Word2Vec integration.

Parameter type	Tested values	Optimal value	Highest coherence score
Vector size	50, 100, 200, 300	200	0.678
Window size	2, 5, 10	2	0.757

With these optimized parameters, the final Word2Vec model was trained and assessed using nearest-neighbor analyses of selected anchor words. The results demonstrated high semantic consistency. For example, the word “speaking” closely associated with “simulated,” “presentation,” and “competence,” and “communication” closely associated with “apprehension,” “language,” and “oral” (Table 2). These strong cosine similarity scores confirm the model’s robust semantic capture within the PSA domain.

**Table 2:** Nearest neighbor evaluation of selected anchor words.

Anchor word	Top related words (cosine similarity ≥ 0.83)
Public	simulated (0.8504), competence (0.8448), oral (0.8372), English (0.8320), learners (0.8319)
Speaking	simulated (0.9161), presentation (0.8938), competence (0.8763), learners (0.8704), fear (0.8702)
Presentation	competence (0.9850), simulated (0.9829), personal (0.9750), speakers (0.9730), speaker (0.9723)
Communication	apprehension (0.9435), language (0.9289), oral (0.9271), English (0.9226), learners (0.9135)

### 3.2 LDA topic modeling results

**Figure 3:**
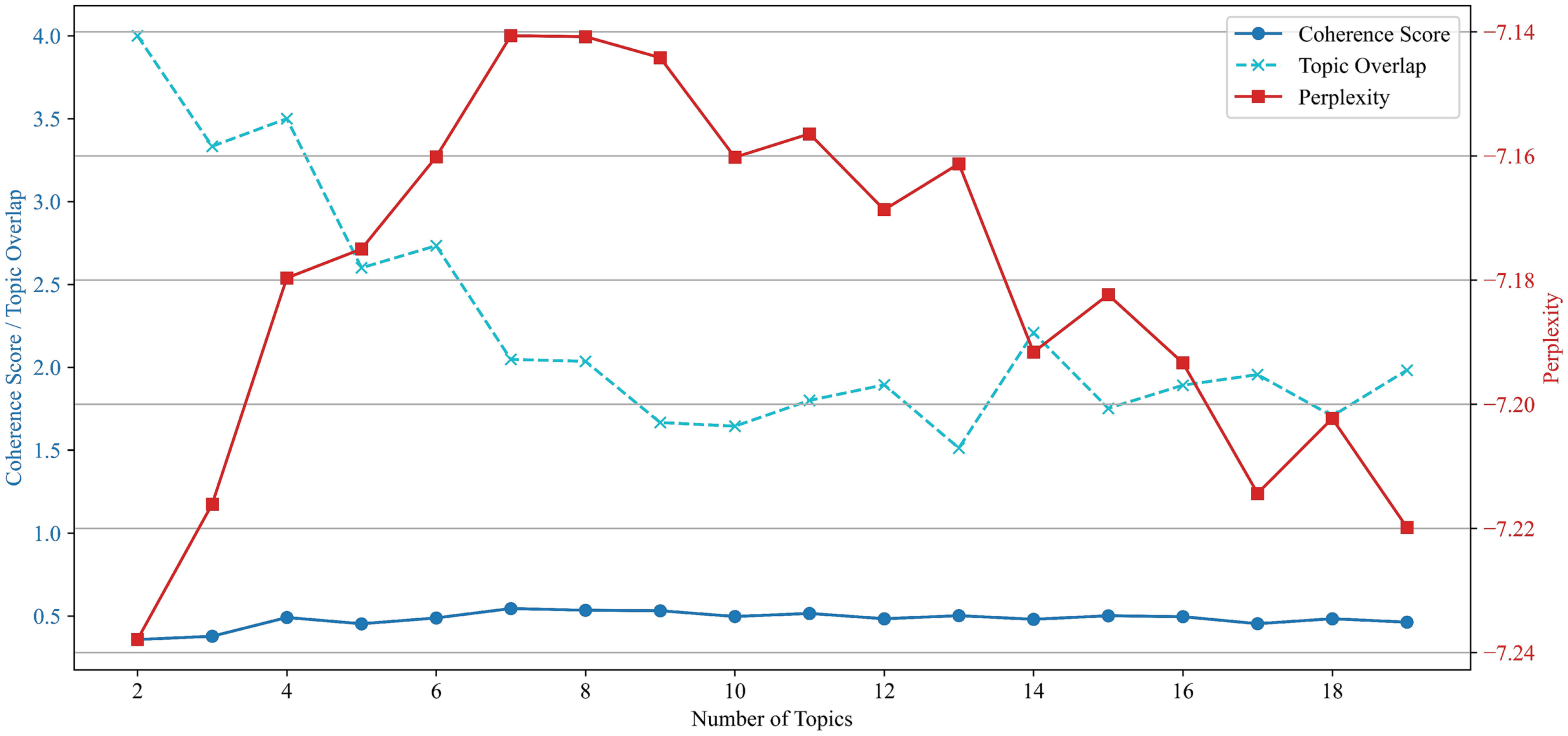
Evaluation of LDA models based on coherence score, perplexity, and topic overlap (2–19 topics).

To identify the underlying thematic structure of public speaking anxiety research, we applied latent Dirichlet allocation (LDA) topic modeling to the cleaned corpus. Multiple candidate models with topic numbers ranging from 2 to 19 were trained and evaluated using three key metrics: coherence score, perplexity, and topic overlap. The results are illustrated in Figure 3.

As shown, topic coherence improved steadily up to seven topics and plateaued thereafter, while perplexity decreased gradually across all settings, indicating improved model fit with more topics. Topic overlap, by contrast, decreased sharply and stabilized between 9 and 13 topics, suggesting increased thematic distinction. Balancing these three indicators, the nine-topic model was selected as optimal, offering a strong trade-off between semantic clarity, statistical fit, and conceptual uniqueness.

Following model selection, the top keywords associated with each topic were extracted and manually interpreted to assign tentative thematic labels. These topics represent a multidimensional view of PSA research, ranging from psychological and physiological responses to educational contexts and virtual exposure therapies. The detailed results are presented in Table 3.

**Table 3:** Extracted topics and associated keywords from LDA analysis.

Topic ID	Label	Top keywords (selected)
0	Pandemic-related mental health	Health, mental, care, pandemic, symptoms, depression, distress, children
1	Speech performance and physiological response	Speech, task, participants, anticipation, physiological, social, response
2	Cortisol-based stress reactivity	Stress, cortisol, response, reactivity, psychological, levels, task
3	Voice anxiety in children	Voice, physical, psychological, mental, anxieties, children, power
4	Communication apprehension in language learning	Students, language, communication, English, learning, oral, apprehension
5	Social phobia and audience-related anxiety	Social, phobia, disorder, performance, fear, attention, participants
6	Virtual exposure and treatment design	Virtual, exposure, treatment, therapy, reality, VR, training, audience
7	Affective and placebo-controlled treatment studies	Effects, placebo, fear, disorder, subjective, treatment, healthy, scale
8	Psychometric validation and measurement reliability	Scale, validity, depression, questionnaire, sample, reliability, scores

**Figure 4:**
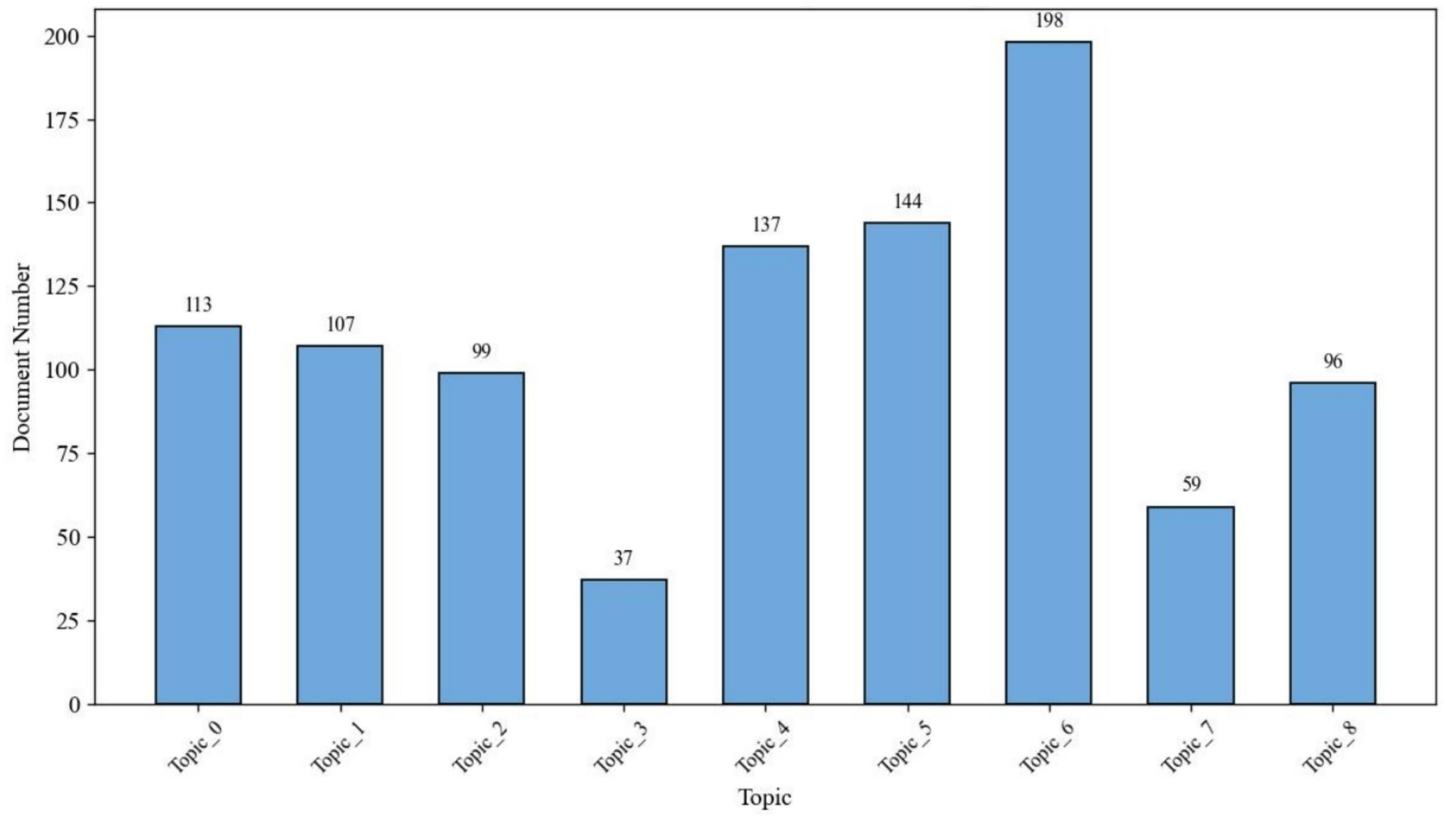
Document distribution by dominant topic.

Additionally, to assess the prevalence of each topic within the corpus, each document was assigned a dominant topic—the one with the highest probability score in its topic distribution. As shown in Figure 4, Topic 6 (Virtual Exposure and Treatment Design) emerged as the most dominant, representing 198 documents. This was followed by Topic 5 (social phobia and audience-related anxiety) and Topic 4 (communication apprehension in language learning), indicating sustained interest in intervention-oriented research and learner-focused anxiety contexts.

This nine-topic framework serves as the analytical foundation for subsequent sections, where thematic clustering, temporal evolution, and emerging research hotspots are analyzed in detail.

### 3.3 LDA-Word2Vec integration results

To enhance thematic representation and support time-aware clustering, the probabilistic topic distributions generated by the LDA model were integrated with semantic word embeddings from the optimized Word2Vec model (as outlined in Section 2.4). Each topic was transformed into a dense semantic vector by computing a weighted average of its top keywords’ Word2Vec embeddings. Similarly, document representations were constructed by aggregating topic vectors weighted by each document’s topic distribution.

Using these semantically enriched document vectors, unsupervised *K*-means clustering was conducted within each of the four pre-defined time periods: 1995–2004, 2005–2013, 2014–2019, and 2020–2024. To determine the optimal number of clusters (*k*) in each stage, silhouette scores were computed across a range of candidate values (*k* = 2 to *k* = 10). Silhouette analysis measures the quality of clustering by evaluating both intra-cluster cohesion and inter-cluster separation.

**Figure 5:**
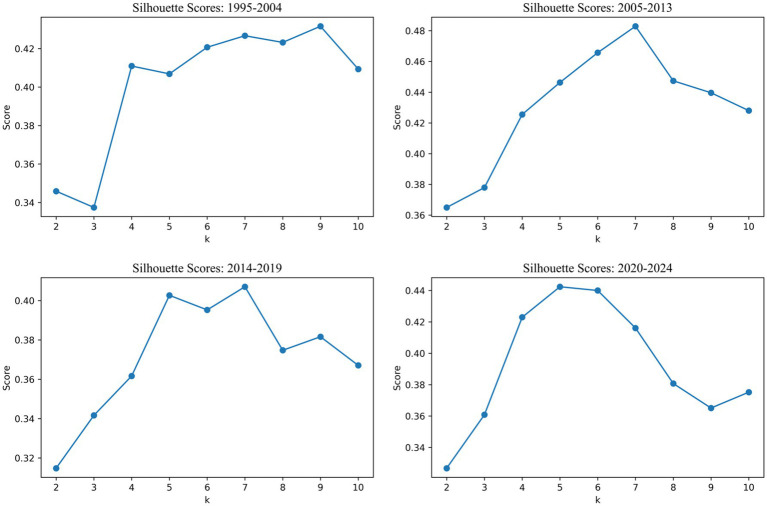
Silhouette scores trends for optimal cluster detection across time periods.

As shown in Figure 5, the silhouette scores varied across time periods, reflecting differences in thematic dispersion and concentration:

- In the 1995–2004 phase, the silhouette score peaked at 0.432 when *k* = 9, suggesting a diverse but separable thematic structure in the early stage of PSA research.
- The 2005–2013 period yielded the highest silhouette value overall (0.483) at *k* = 7, indicating clearly delineated clusters during this era of thematic expansion.
- For 2014–2019, the optimal *k* remained at 7, but the maximum silhouette score dropped slightly to 0.407, reflecting increasing thematic overlap and interdisciplinary blending.
- In the most recent stage, 2020–2024, the optimal number of clusters decreased to *k* = 5, with a silhouette score of 0.442, suggesting a trend toward thematic convergence around fewer, more cohesive areas of research.

The clustering outputs not only offer insights into how PSA research was organized within each historical phase, but also reveal the dynamic nature of thematic consolidation over time. A larger number of clusters in earlier phases (e.g., nine in 1995–2004) corresponded to a wider dispersion of research topics, whereas the more recent periods featured fewer clusters and stronger cohesion, suggesting greater conceptual convergence.

To interpret the content of each cluster, dominant topics were identified based on the highest average topic weight among member documents. The results are summarized in Table 4, which lists the dominant topics of each cluster alongside their mean topic probabilities.

**Table 4:** Distribution of research topics by cluster and period.

Time period	Cluster number	Cluster dominant topics
1995–2004	9	Cluster 0—Topic_0 (0.398), Cluster 1—Topic_1 (0.669), Cluster 2—Topic_6 (0.504), Cluster 3—Topic_6 (0.892), Cluster 4—Topic_2 (0.803), Cluster 5—Topic_5 (0.823), Cluster 6—Topic_8 (0.471), Cluster 7—Topic_4 (0.703), Cluster 8—Topic_7 (0.699)
2005–2013	7	Cluster 0—Topic_0 (0.428), Cluster 1—Topic_2 (0.795), Cluster 2—Topic_5 (0.653), Cluster 3—Topic_1 (0.565), Cluster 4—Topic_6 (0.740), Cluster 5—Topic_8 (0.699), Cluster 6—Topic_4 (0.664)
2014–2019	7	Cluster 0—Topic_4 (0.593), Cluster 1—Topic_8 (0.621), Cluster 2—Topic_2 (0.640), Cluster 3—Topic_6 (0.709), Cluster 4—Topic_5 (0.761), Cluster 5—Topic_0 (0.518), Cluster 6—Topic_1 (0.365)
2020–2024	5	Cluster 0—Topic_0 (0.572), Cluster 1—Topic_6 (0.639), Cluster 2—Topic_8 (0.341), Cluster 3—Topic_2 (0.506), Cluster 4—Topic_4 (0.671)

These clustering results reflect the evolving thematic configuration of PSA research over the past three decades. Earlier phases were characterized by a relatively higher number of clusters, suggesting a broader and more fragmented research landscape. In contrast, more recent phases exhibited fewer but more cohesive clusters, indicating increasing thematic consolidation. This temporal trend suggests a maturing field, where research directions are progressively converging around stable, semantically coherent topic areas.

The identified cluster-topic structures in each phase lay the foundation for mapping longitudinal transitions across time segments, which will be further examined in the following section on topic evolution dynamics.

## 4 Discussion

### 4.1 Identification of research hotspots across time segments

**Figure 6:**
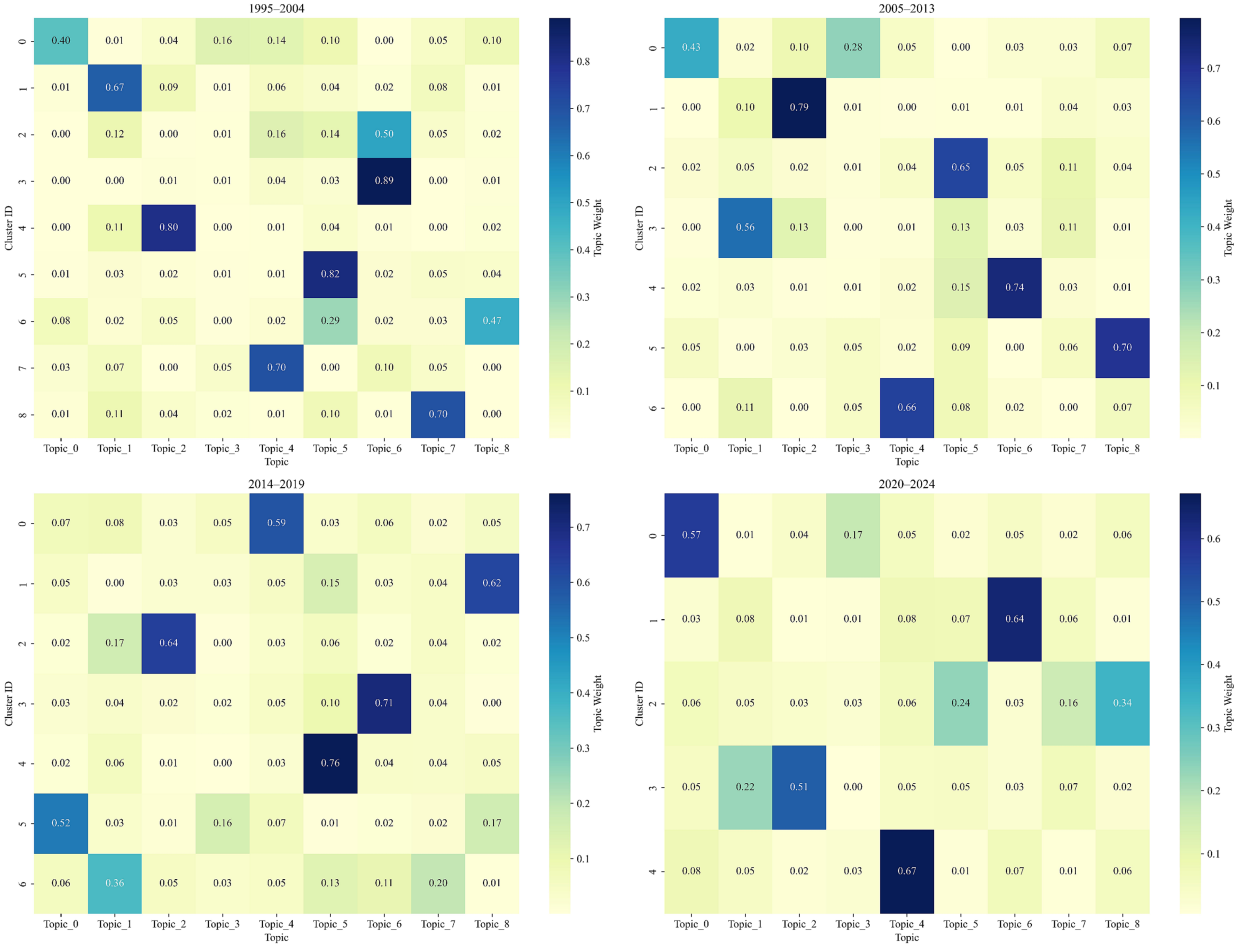
Topic intensity across time segments.

To explore how key research themes evolved within different temporal stages of public speaking anxiety (PSA) scholarship, this study examined the distribution of topics across document clusters in four chronological segments: 1995–2004, 2005–2013, 2014–2019, and 2020–2024. The analysis relied on topic-cluster matrices generated through the fusion of LDA topic distributions with Word2Vec-enhanced semantic vectors. Each matrix captured the average topic weight within each cluster, thereby highlighting the most prominent themes characterizing each period. Figure 6 presents a consolidated heatmap displaying the intensity of topic presence across all clusters and phases. Darker shades indicate higher average topic weights, signifying greater thematic dominance within a given cluster.

#### 4.1.1 1995–2004: diverse but fragmented early themes

During the earliest stage of PSA research (1995–2004), the corpus was partitioned into nine distinct clusters, each characterized by a dominant topic with varying degrees of concentration (see Figure 6). This phase displayed a broad and relatively fragmented thematic structure, underscoring the exploratory nature of early scholarship in the field.

Two clusters—Cluster 2 and Cluster 3—were both dominated by Topic 6 (Virtual Exposure and Treatment Design), but with distinct semantic emphases. Cluster 3 exhibited a highly concentrated thematic structure, with Topic 6 accounting for 89.2% of its topic distribution, suggesting a tight focus on experimental interventions and virtual reality-based exposure treatments. In contrast, Cluster 2—while also led by Topic 6 (50.4%)—demonstrated a more multifaceted composition, incorporating secondary contributions from Topic 4 (communication apprehension) at 16.1%, Topic 5 (social phobia and performance anxiety) at 13.7%, and Topic 1 (speech task and physiological response) at 11.7%. This indicates that research in Cluster 2 explored more complex or interdisciplinary settings, such as the use of virtual exposure in student language-learning contexts or in conjunction with physiological outcome measures.

Elsewhere, Cluster 5 was defined by Topic 5, with a dominant weight of 82.3%, highlighting the early focus on clinical constructs such as social anxiety disorder and fear of performance. Cluster 4 focused on Topic 2 (Cortisol-Based Stress Reactivity) with a similarly high dominance of 80.3%, reflecting the incorporation of physiological and neuroendocrine measures into PSA assessments.

In the education-focused strand of research, Cluster 7 prominently featured Topic 4, associated with communication apprehension in second-language learning, holding a weight of 70.3%. Additionally, Cluster 1 was defined by Topic 1, involving anticipatory physiological responses during speech tasks, further illustrating methodological diversity in early-stage PSA studies.

Taken together, this period reflects a field in formation, marked by a broad array of epistemological approaches—from psychophysiology and clinical interventions to educational psychology. However, the large number of clusters and the dispersion of topics also point to a lack of thematic consolidation, suggesting that PSA was still emerging as a coherent domain of inquiry during this stage. Notably, the prominence of communication apprehension in second-language learning (e.g., Cluster 7) aligns with communication apprehension theory (McCroskey, 1970), which conceptualizes communication anxiety as a trait-like or situational factor shaping individuals’ willingness to communicate. Early studies of PSA mirrored this perspective by embedding communication apprehension in educational contexts, thereby anticipating later findings that high levels of apprehension undermine oral proficiency and academic engagement (Bodie, 2010). More recent extensions of the theory into digital and hybrid classrooms (Molnar and Crnjak, 2018; Agrawal and Krishna, 2021) confirm that the fragmented early focus on communication apprehension laid a foundation for subsequent explorations of speech anxiety across multimodal and technology-mediated environments.

#### 4.1.2 2005–2013: toward thematic consolidation and clinical emphasis

In the second phase (2005–2013), PSA research began to exhibit signs of thematic consolidation, with seven clusters identified—fewer than in the previous phase—indicating a more focused and stable research structure. The average silhouette score for this phase was relatively high (e.g., reaching 0.420 at *k* = 7), suggesting improved cluster cohesion and inter-cluster separability compared to the exploratory dynamics of the prior decade.

Three clusters stood out for their strong topic dominance and clear thematic identity:

- Cluster 1 was overwhelmingly driven by Topic 2 (cortisol-based stress reactivity) at 79.5%, continuing the psychophysiological trajectory from the previous period but with clearer boundaries and a more specialized focus.
- Cluster 4 featured Topic 6 (virtual exposure and treatment design) with a high weight of 74.0%, signifying the growing academic and clinical interest in virtual reality-based interventions for PSA.
- Cluster 2 centered on Topic 5 (social phobia and performance anxiety) with a 65.3% share, indicating a persistent clinical orientation focused on the psychological underpinnings of performance-related anxiety.

Other clusters revealed a more interdisciplinary expansion:

- Cluster 0 involved Topic 0 (mental health and pandemic-related distress) at 42.8%, but also incorporated Topic 3 (voice anxiety and psychosocial factors) and Topic 8 (measurement and psychometrics). This blend implies a movement toward integrating broader mental health concerns and instrument validation in PSA research.
- Cluster 3, led by Topic 1 (speech task and physiological response) at 56.5%, reflected the sustained importance of lab-based task paradigms in measuring PSA responses.
- Cluster 6, where Topic 4 (communication apprehension) held 66.4%, continued to represent second-language education and student-centered communication studies.

This period marked a shift from fragmented exploration to structured knowledge-building, with clusters demonstrating clearer topical boundaries and more refined methodological orientations. In particular, the prominence of virtual exposure and treatment design (Cluster 4) reflects Social Cognitive Theory’s emphasis on shaping behavior through mastery experiences and modeling, whereby repeated exposure to simulated speaking contexts builds self-efficacy and reduces anxiety (Bandura, 1986). Recent empirical studies, including gamified VR exposure (Kahlon et al., 2023) and co-regulated collaborative speaking tasks (Hao and Chen, 2024), confirm that modeled or mediated experiences significantly bolster public speaking self-efficacy and attenuate PSA. The increased dominance scores suggest that research communities were converging around well-defined problems, particularly in clinical and experimental domains. Moreover, the persistence of key topics from the previous phase—now appearing in more distinct clusters—signals the maturation and specification of PSA research, laying a stable groundwork for future interdisciplinary synthesis.

#### 4.1.3 2014–2019: accelerated expansion and interdisciplinary integration

The third stage (2014–2019) witnessed a period of accelerated thematic expansion and interdisciplinary convergence, as evidenced by the continued presence of seven clusters and elevated silhouette scores. The optimal clustering configuration (*k* = 7) yielded a silhouette score of 0.441, reflecting increasingly well-defined topical groupings and a stable knowledge structure.

This period retained several dominant clusters from earlier stages, but also introduced new interdisciplinary linkages and greater semantic overlap among themes. Notable clusters include:

- Cluster 4, led by Topic 5 (social phobia and performance anxiety) with a 76.1% weight, continued to occupy a central position in PSA research. Its persistence across multiple stages highlights the sustained importance of clinical and diagnostic approaches.
- Cluster 3, dominated by Topic 6 (virtual exposure and treatment design) at 70.9%, underscores the consolidation of virtual reality (VR) and simulation-based therapy as a mature subfield. Compared to the previous phase, this topic appears more integrated into experimental design and intervention studies.
- Cluster 2, with Topic 2 (cortisol-based stress reactivity) accounting for 64.0%, reaffirms the relevance of psychophysiological metrics, though the thematic scope appears to narrow compared to its previous representations.

Importantly, newly emphasized dimensions emerge in other clusters:

- Cluster 0, with Topic 4 (communication apprehension and second-language anxiety) at 59.3%, reflects growing attention to PSA in multilingual and multicultural learning environments, particularly among university students and ESL learners.
- Cluster 1, with Topic 8 (measurement and psychometrics) taking 62.1%, highlights a methodological turn toward scale development, validation, and measurement precision—critical for advancing empirical rigor.
- Cluster 6, where Topic 1 (speech task and anticipatory response) held 36.5%, reveals the declining dominance of this once-prevalent theme, possibly due to saturation or integration into broader constructs like social phobia.
- Cluster 5, with Topic 0 (mental health and pandemic-related distress) as the core at 51.8%, may reflect early intersections with global health concerns, though its prominence would surge more visibly in the following phase.

Amid this expansion, the prominence of topics supporting learner agency and methodological rigor—specifically, clusters addressing multilingual communication anxiety (Topic 4) and measurement development (Topic 8)—resonate strongly with self-determination theory (Deci and Ryan, 2000). Supporting this theoretical alignment, a recent meta-review of SDT-based interventions in educational domains confirmed their effectiveness in enhancing autonomy, competence, and psychological wellbeing (Wang et al., 2024).

This phase demonstrates a notable thematic branching: while core clinical topics remain strong, newer subfields—particularly psychometrics, educational linguistics, and VR-based interventions—gain visibility and intellectual autonomy. Clusters in this phase show greater topic interpenetration, indicating a shift toward interdisciplinary integration and methodological diversification, positioning PSA research as a richly layered and rapidly evolving field.

#### 4.1.4 2020–2024: intensified focus and emerging hybrid themes

The final period (2020–2024) marked a phase of thematic consolidation coupled with emerging hybrid orientations, as shown by the reduced number of clusters (*k* = 5) and the highest silhouette score of 0.466 among all phases. This indicates both sharper intra-cluster cohesion and stronger inter-cluster differentiation, reflecting a maturing field with increasingly specialized research directions.

Several key trends are observable:

- Cluster 1, dominated by Topic 6 (virtual exposure and treatment design) with a weight of 63.9%, stands out as a major thematic anchor, highlighting the widespread integration of VR technologies and online interventions. This trend reflects the post-pandemic surge in digital therapy formats and remote exposure programs for PSA management.
- Cluster 4, led by Topic 4 (communication apprehension and second-language anxiety) at 67.1%, continues its rise as a mainstream concern, especially in education and applied linguistics. The consistent growth of this topic since 1995 suggests its entrenchment in globalized academic discourse and the increased attention to anxiety in ESL/EFL contexts.
- Cluster 0, driven by Topic 0 (mental health and pandemic-related distress) at 57.2%, exemplifies the emergence of hybrid research linking PSA to broader public health crises, such as COVID-19-induced anxiety, depression, and psychological distress. Its presence underscores a shift from isolated speech anxiety to systemic affective dynamics within larger societal disruptions.
- Cluster 3, where Topic 2 (cortisol-based stress reactivity) holds 50.6%, indicates sustained interest in physiological and endocrine correlates of PSA, often used to validate behavioral or therapeutic findings. Compared to earlier phases, however, this theme seems less central, hinting at possible methodological plateauing.
- Cluster 2, despite having the lowest dominant topic weight at 34.1% (Topic 8: measurement and psychometrics), points toward growing complexity and multi-thematic entanglement. The lower concentration suggests a transition zone or experimental arena where no single theme dominates, potentially signaling emerging or hybrid topics not yet fully crystallized.

Overall, this period is characterized by greater depth, specificity, and responsiveness to real-world challenges, particularly the COVID-19 pandemic and technological acceleration. The rise of dominant digital, linguistic, and hybrid health-focused clusters reflects a thematic refinement, while the appearance of weaker clusters hints at incipient interdisciplinary syntheses. The balance between high silhouette cohesion and reduced cluster quantity confirms the intellectual consolidation of PSA research, now transitioning into a post-mature stage marked by specialization and convergence. Notably, the increasing attention to post-pandemic mental health, multicultural learning contexts, and adaptive digital interventions resonates with anxiety/uncertainty management theory (AUM), which emphasizes the regulation of uncertainty and anxiety in communication. By highlighting how speakers manage ambiguity and emotional stress in intercultural or crisis settings, AUM provides a valuable lens for understanding PSA research’s recent orientation toward context-sensitive, technologically mediated, and culturally adaptive approaches (Gudykunst, 2005).

### 4.2 Dynamic evolution of research topics

To capture the longitudinal dynamics of public speaking anxiety (PSA) research, a semantic-cluster-based topic evolution map was constructed by integrating latent Dirichlet allocation (LDA) with Word2Vec-based document embeddings. The method enabled both intra-period clustering and inter-period semantic comparison. Each cluster was characterized by a dominant topic determined by averaging the LDA topic probabilities of its member documents. Inter-cluster connections across adjacent time periods were computed using cosine similarity between mean vector representations, with a threshold set at 0.8 to retain only semantically robust transitions.

**Figure 7:**
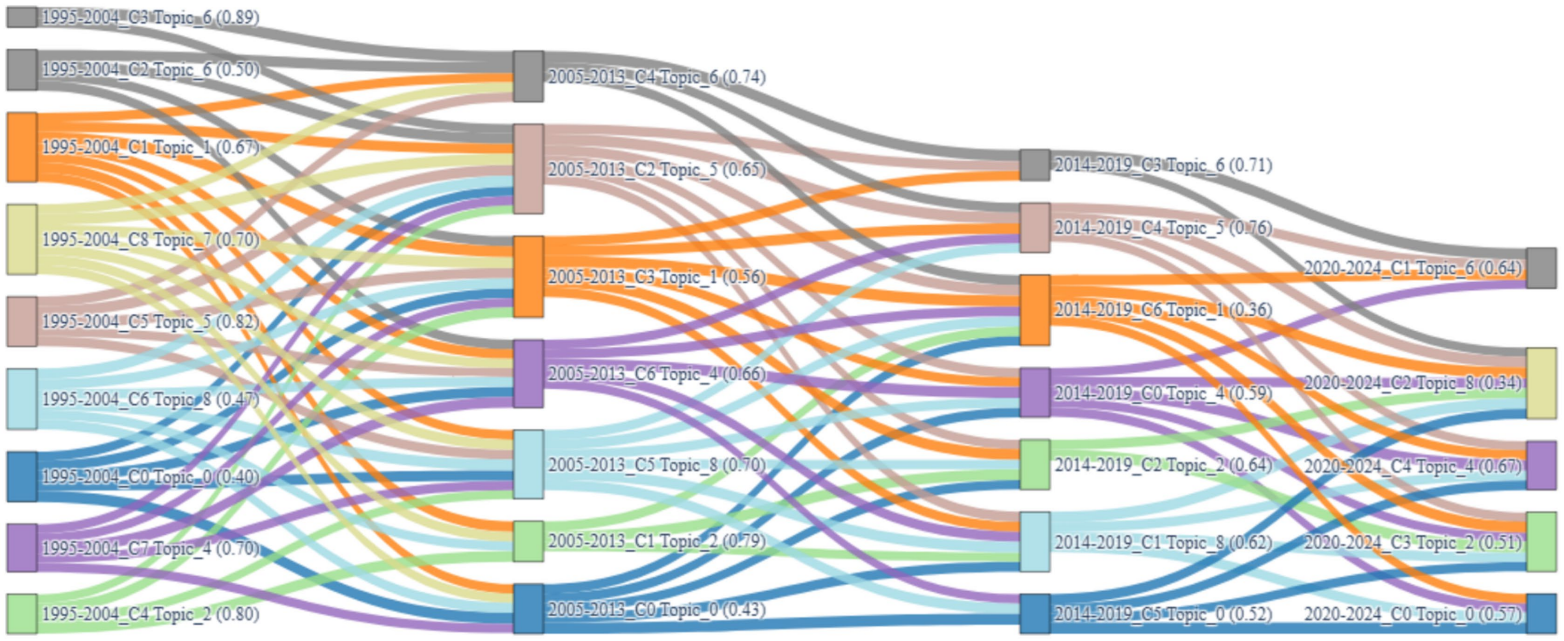
Sankey diagram of topic cluster evolution (1995–2024).

Figure 7 presents a Sankey diagram that visualizes these topic transitions across the four temporal segments (1995–2004, 2005–2013, 2014–2019, and 2020–2024). Each node represents a topic-dominant cluster in a specific phase, color-coded by its dominant topic. The thickness of the connecting lines indicates the degree of semantic continuity, derived from cosine similarity between cluster centers. This visualization allows for a simultaneous assessment of three dimensions: continuity, strength, and topic-level evolution pathways.

#### 4.2.1 Temporal continuity and shifts of dominant themes

The diagram reveals that certain topics, particularly Topic_6 (e.g., “virtual,” “exposure,” “treatment,” “therapy”) and Topic_4 (e.g., “students,” “language,” “communication,” “oral”), maintain consistent dominance across multiple stages. Topic_6 appears as the dominant theme in Cluster 2 and Cluster 3 during 1995–2004, with weights of 0.504 and 0.892, respectively, and continues through Cluster 4 in 2005–2013 (0.740), Cluster 3 in 2014–2019 (0.709), and Cluster 1 in 2020–2024 (0.639). Its recurrent role and multiple thick connections in the Sankey plot signify a robust evolutionary path.

In contrast, Topic_0 (e.g., “health,” “mental,” “pandemic”) surfaces only in the most recent phase (2020–2024, Cluster 0, 0.572), reflecting a responsive emergence aligned with the COVID-19 pandemic. Its isolated position in the Sankey diagram—without incoming or outgoing links—suggests a novel yet disconnected thematic insertion.

#### 4.2.2 Strength of evolution and transition patterns

The relative thickness and density of linkages between nodes in Figure 7 indicate the strength of topic evolution. Topic_6 leads with the highest number of retained transitions (five links across three time segments), highlighting its semantic resilience and adaptability. Topic_4 follows, connecting through Cluster 7 (1995–2004), Cluster 6 (2005–2013), Cluster 0 (2014–2019), and Cluster 4 (2020–2024), with dominance scores ranging from 0.593 to 0.703.

In contrast, themes such as Topic_3 (e.g., “voice,” “psychological,” “power”) and Topic_7 (e.g., “effects,” “subjective,” “treatment”) show weak or nonexistent transitions, represented in Figure 7 by their terminal positions with no outgoing or incoming edges. These themes can be considered phase-specific or auxiliary in nature.

#### 4.2.3 Functional typology of evolving themes

Based on visual and quantitative evidence from Figure 7 and cluster-topic dominance matrices, topics can be grouped as follows:

- Stable Core Themes: Topic_6 and Topic_4, which exhibit multi-stage transitions, strong inter-cluster continuity, and repeated dominance (≥0.5) in each phase.
- Transient or Event-Driven Themes: Topic_0 and Topic_3, which emerge in response to specific contexts (e.g., health crises or media shifts), as evidenced by isolated node positions and lack of linkage in Figure 7.
- Peripheral Support Themes: Topic_2 (stress physiology), Topic_5 (social phobia), and Topic_8 (psychometrics), which appear consistently but with lower dominance and weaker transitions.

This structured typology, reinforced by the Sankey diagram’s temporal linkage logic and vector-based computation, provides a nuanced understanding of the thematic life cycle in PSA research.

### 4.3 Predicting future research hotspots in public speaking anxiety

To identify and evaluate emerging trends within the PSA literature, this study introduces two novelty-oriented indicators: Cluster Novelty and Topic Novelty. Unlike raw publication counts, which merely reflect volume, these indicators leverage the temporal distribution of documents to capture how recently specific clusters or subtopics have been actively developed. In other words, novelty is not a measure of size but of recency-weighted activity, distinguishing between themes sustained by older studies and those energized by new contributions. For instance, two clusters with similar publication volumes may diverge sharply in novelty if one is anchored in earlier decades while the other is driven by recent work. By quantifying such differences, novelty scores provide a more fine-grained lens to assess thematic vitality, intellectual momentum, and the likelihood of future expansion.

#### 4.3.1 Cluster novelty

Cluster Novelty is defined as the average publication year of all documents grouped under a specific cluster. It reflects the temporal recency of a cluster as a whole, thereby indicating its potential to represent cutting-edge or emerging research directions. Mathematically, it is calculated as:


$$N_{C}=\frac{1}{n}\sum_{i=1}^{n_{c}}y_{i}$$

where $N_{C}\;$ denotes the novelty score of cluster c ; $y_{i}$ is the publication year of document i within cluster c ; $n_{c}$ is the total number of documents assigned to cluster c .

A higher $N_{C}$ value (closer to 2024) suggests that the cluster contains more recently published articles, indicating stronger alignment with current or emerging research interests.

As shown in Table 5, Cluster C4 (Topic_4), and Cluster C1 (Topic_6) attain the highest novelty scores of 2022.326 and 2022.225, respectively, signifying their potential as contemporary research frontiers. These clusters are associated with themes such as communication apprehension in students and virtual exposure therapy, both of which have grown rapidly in recent years.

**Table 5:** Cluster novelty scores and dominant topics (2020–2024).

Cluster label	Average year (novelty score)	Dominant topic
2020–2024_C4	2022.326	Topic_4
2020–2024_C1	2022.225	Topic_6
2020–2024_C2	2022.111	Topic_7
2020–2024_C0	2021.827	Topic_0
2020–2024_C3	2021.536	Topic_2

These results align closely with prior trend analyses (Section 4.2), where Topic_4 (“students, language, communication, oral”) and Topic_6 (“virtual, exposure, therapy, audience”) were shown to exhibit strong temporal continuity and adaptive potential.

#### 4.3.2 Topic novelty within clusters

Topic Novelty further examines the internal composition of each cluster by calculating the weighted average publication year of documents associated with each topic. The $N_{c,t}$ score for topic t in cluster c is defined as:


$$N_{c,t}=\frac{1}{n_{c,t}}\sum_{i=1}^{n_{c,t}}y_{i}$$

where $y_{i}\;$ is the publication year of document i , and $n_{c,t}$ is the number of documents in cluster c that include topic t . This unweighted mean reflects the temporal freshness of a topic within each thematic cluster and enables intuitive comparison across topics and time segments.

**Figure 8:**
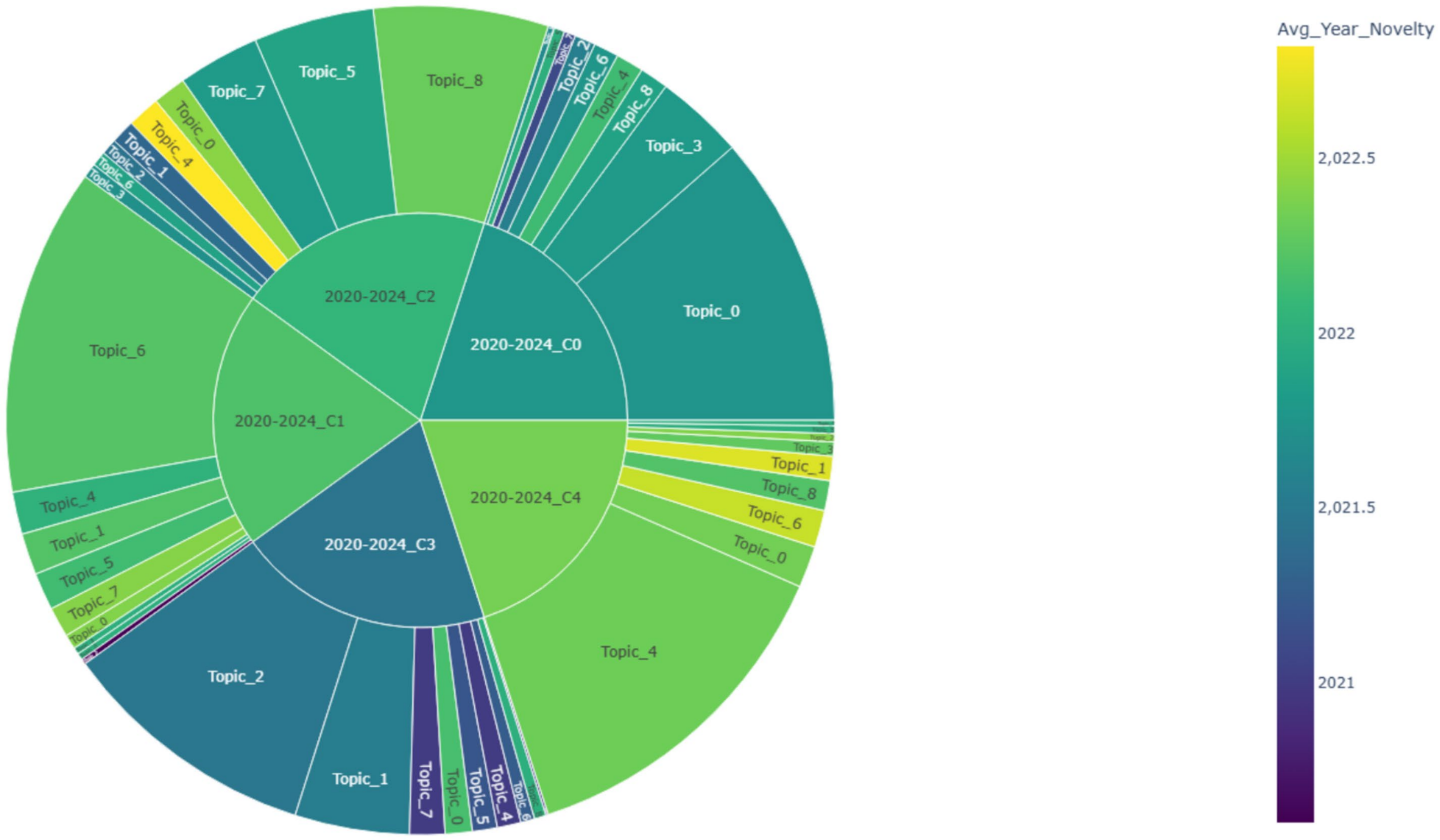
Sunburst visualization of topic novelty and weight within clusters (2020–2024).

To visualize the distribution of topic novelty within clusters, a hierarchical sunburst diagram was constructed (Figure 8). The inner ring represents five clusters derived from the 2020–2024 phase, and the outer ring shows the topics associated with each cluster. The area of each wedge is proportional to the topic’s weight (i.e., its relative contribution to the cluster), while color indicates average publication year. Lighter tones (toward yellow) correspond to higher novelty, whereas darker hues (toward blue) suggest older research activity.

From the visualization, Cluster C4 demonstrates the highest concentration of novel topics, with Topic 4 (language learning and communication apprehension) occupying the largest area and displaying a relatively bright color (Avg. Year ≈ 2022.33). Similarly, Topic 6 and Topic 1 also appear prominently in C4, indicating this cluster’s strong association with recently emerging directions in educational and physiological approaches to public speaking anxiety.

Cluster C1, while slightly less novel on average, is still characterized by the dominance of Topic 6 (virtual reality exposure therapy) and noticeable contributions from Topics 4, 5, and 7, all of which exhibit average years above 2022.2, suggesting robust novelty. In contrast, Cluster C3 is dominated by Topic 2 (stress and cortisol response), with a much lower novelty score (≈ 2021.48), visualized by its darker shade and larger size—signifying both maturity and conceptual stability.

Taken together, the sunburst diagram offers a dual-lens perspective: it not only highlights emerging themes (e.g., Topics 4, 6, and 8) but also reveals their distribution and salience across thematic clusters. This enables scholars to pinpoint not only which topics are gaining traction, but also where they are contextually embedded within broader research trends.

#### 4.3.3 Predictive validity of novelty indicators

To further validate the effectiveness of the novelty metrics, we implemented a backtesting procedure across consecutive time segments. Specifically, cluster- and topic-level novelty scores were computed for one period (e.g., 1995–2004), and the resulting set of predicted “novel” topics was compared against the actual dominant topics that emerged in the subsequent period (2005–2013). Predictive performance was quantified using precision (proportion of predicted topics that actually appeared later) and recall (proportion of actual topics that were successfully anticipated).

The results (Table 6) show that in the first two backtests (1995–2004 → 2005–2013; 2005–2013 → 2014–2019), novelty scores achieved near-perfect predictive power (precision ≥ 0.875; recall = 1.0). The combination of perfect recall with slightly lower precision indicates that the indicators were highly sensitive to the detection of all emerging topics, but also identified a small number of “false positives.” These false positives typically correspond to themes that exhibited short-lived novelty in one period but failed to develop into dominant clusters in the next. While this reduces precision, it reflects an acceptable trade-off for horizon scanning, where minimizing the risk of missing genuine emerging topics (high recall) is often prioritized over absolute precision.

**Table 6:** Backtesting results of novelty indicators.

Training period	Testing period	Precision	Recall
1995–2004	2005–2013	0.875	1.000
2005–2013	2014–2019	1.000	1.000
2014–2019	2020–2024	0.571	0.800

In the second backtest (2005–2013 → 2014–2019), both precision and recall reached 1.000. This apparent “perfect prediction” is not evidence of overfitting but rather a reflection of the clear continuity and consolidation of research trajectories during this stage. Because novelty scoring was strictly based on temporally segmented data, with no access to subsequent periods, the process avoided retrospective bias or data leakage. The perfect match emerged as a consequence of the stable thematic transition between these two periods, rather than any methodological artifact.

In the final test (2014–2019 → 2020–2024), predictive accuracy declined (precision = 0.571, recall = 0.800), reflecting the disruptive emergence of hybrid themes in the COVID-19 era. Importantly, this decline underscores the sensitivity of novelty indicators to unforeseen but significant paradigm shifts, reinforcing their value for distinguishing between continuing themes and truly emergent directions.

#### 4.3.4 Research implications

The combined analysis of cluster- and topic-level novelty offers critical insights for anticipating emerging frontiers in public speaking anxiety (PSA) research. Several implications can be drawn.

First, clusters such as C4 and C1, which exhibit both high average publication years and dominant topics with strong novelty signals (e.g., Topic 4: language learning and communication apprehension, and Topic 6: virtual therapy and technological interventions), represent likely trajectories of future development. These clusters reveal a notable shift from traditional stress-oriented models to technology-enhanced, learner-centered, and educationally integrated frameworks. Such transitions reflect growing attention to PSA in virtual, multilingual, and hybrid learning contexts—particularly in response to remote instruction and online communication challenges post-COVID-19.

Second, the presence of Topic 8 (psychometric validation and assessment tools) with rising novelty in both C2 and C4 suggests increasing methodological sophistication. This trend highlights the field’s movement toward more nuanced, scalable, and empirically validated instruments for measuring anxiety and performance-related constructs. Future research may further refine cross-cultural scales, real-time monitoring methods, and interdisciplinary evaluation tools, integrating cognitive, affective, and technological dimensions.

Third, while certain clusters (e.g., C0 and C3) are driven by relatively mature themes such as Topic 0 (pandemic mental health) and Topic 2 (cortisol and physiological response), their declining novelty scores suggest possible thematic saturation. However, their continued presence indicates that these domains still serve as foundational anchors, offering theoretical stability and interdisciplinary bridges with psychology, psychiatry, and biomedical sciences.

Finally, the methodological framework proposed in this study—integrating LDA, Word2Vec, cluster-based evolution modeling, and novelty forecasting—provides a transferable analytical paradigm. This approach can be adapted to other domains where long-term thematic mapping, semantic enrichment, and research horizon scanning are critical.

In sum, the findings underscore a multidimensional shift in PSA research toward greater contextual diversity, technological embedding, and learner-specific focus, thereby charting an evidence-based roadmap for future inquiry.

## 5 Conclusion

### 5.1 Summary of key findings

This study provides a comprehensive bibliometric and semantic analysis of public speaking anxiety (PSA) research spanning from 1995 to 2024. Through the use of latent Dirichlet allocation (LDA) topic modeling, nine major thematic clusters were identified, capturing both traditional and emerging areas of inquiry within the field. These include mental health and pandemic-related distress, anticipatory physiological responses during speech tasks, cortisol-based stress reactivity, vocal and psychological anxieties, communication apprehension in language learning, social phobia and performance anxiety, virtual reality-based therapy, placebo-controlled treatment effects, and psychometric validation. Collectively, these themes reflect a field that balances classical psychological foundations with contemporary technological and educational innovations.

Second, by integrating Word2Vec-based semantic embeddings with LDA-derived topic distributions, the study developed enriched document and topic vectors, allowing for more nuanced clustering and temporal mapping. This fusion enabled the segmentation of the PSA research landscape into four evolutionary phases: the Emergent Phase (1995–2004), marked by limited output and fragmented themes; the Early Expansion Phase (2005–2013), characterized by increased empirical and clinical attention; the Consolidation Phase (2014–2019), which saw greater thematic diversification and interdisciplinary integration; and the High Productivity Phase (2020–2024), defined by rapid growth, heightened attention to digital communication anxiety, and growing emphasis on measurement tools and remote interventions.

Third, the analysis of topic continuity and transformation using vector-based similarity and Sankey visualization revealed distinct thematic trajectories. While topics such as virtual exposure therapy and physiological stress responses showed sustained presence across phases, others emerged in response to contextual triggers or declined over time. To further identify potential research frontiers, the study introduced two novelty-based indicators: Cluster Novelty, reflecting the average publication year of documents within a cluster, and Topic Novelty, measuring the recency of individual topics embedded in clusters. The results highlighted Clusters C4, C1, and C2—dominated by Topic 4 (language learning and communication apprehension), Topic 6 (virtual therapy), and Topic 8 (psychometric assessment), respectively—as the most temporally novel, indicating strong momentum toward technology-driven, education-focused, and empirically grounded approaches in PSA research.

### 5.2 Implications and future directions

The findings of this study have several important implications for future PSA scholarship. First, the continued rise of themes such as virtual intervention, language learning anxiety, and online communication competence suggests a field increasingly attuned to digital transformation and hybrid learning environments. This trajectory resonates with communication apprehension theory, which conceptualizes PSA as rooted in situational, individual, and contextual factors that affect communicative performance (McCroskey, 1977, 1984). Future research should extend this theoretical foundation to remote and multilingual contexts, exploring the efficacy of VR- or AI-assisted training systems that adapt to individual differences in speech anxiety.

Second, the growing visibility of psychometric validation and assessment tools reflects an ongoing refinement of PSA measurement practices. This aligns with Social Cognitive Theory, which emphasizes reciprocal determinism between personal, behavioral, and environmental factors (Bandura, 1986, 1999). Future studies may therefore design real-time, scalable, and cross-culturally adaptive instruments that integrate cognitive, affective, and physiological data streams. Such tools would not only advance methodological rigor but also enhance intervention design in both educational and clinical settings.

Third, the persistence of physiological stress responses and pandemic-related anxieties in lower-novelty clusters suggests that these domains remain conceptual anchors. Their relevance can be illuminated through Self-Determination Theory, which highlights autonomy, competence, and relatedness as basic psychological needs influencing motivation and stress regulation (Deci and Ryan, 2000, 2013). Future research might build on this perspective to create supportive learning and therapeutic environments that not only mitigate PSA but also promote resilience and intrinsic motivation in speakers.

Finally, the methodological framework proposed in this study—integrating topic modeling, word embeddings, temporal segmentation, and novelty metrics—proved not only flexible and replicable but also empirically robust. Through backtesting across sequential time periods, the novelty-based indicators demonstrated their ability to anticipate topic continuity, providing preliminary evidence of predictive validity. This strengthens their value as tools for horizon scanning in PSA research and underscores their potential adaptability to other domains, such as education, health communication, and psychological intervention research, where forecasting thematic trajectories and mapping emerging knowledge frontiers are equally essential.

### 5.3 Strengths and limitations

The primary strength of this study lies in its integrated methodological design, which combines topic modeling and word embedding to reveal both statistical and semantic layers of scholarly discourse. This hybrid framework enables high-resolution analyses of thematic evolution, cluster-based transitions, and trend prediction. Importantly, the predictive capacity of the novelty indicators was not only conceptually motivated but also empirically validated through backtesting, which confirmed their ability to capture forward-looking thematic dynamics. The use of cluster- and topic-level novelty metrics provides a theoretically grounded and quantitatively interpretable approach for forecasting future hotspots in PSA research. Furthermore, the chronological segmentation of data adds temporal granularity to the findings, facilitating a more structured interpretation of research trends.

However, certain limitations warrant acknowledgment. First, the corpus is restricted to English-language journal articles indexed in the Web of Science, potentially excluding valuable non-English or regionally specific literature, as well as gray literature. This reliance may limit the global representativeness of the findings. Second, the manual assignment of topic labels, while guided by keyword distributions, inevitably introduces a degree of subjectivity. Although we cross-validated labels with domain expertise, no formal inter-coder agreement statistics were reported, which should be considered in future studies to strengthen reliability. Third, while the combination of LDA and Word2Vec improves semantic granularity, it introduces additional complexity and sensitivity to parameter tuning (e.g., number of topics, alpha/beta priors, Word2Vec dimensions, and window size). Although coherence-based validation was applied, variations in parameterization may affect replicability across datasets. Fourth, although novelty indicators achieved strong predictive performance, their tendency toward high recall but lower precision implies a trade-off: the framework is highly sensitive in detecting potential emerging topics, but may also flag short-lived signals that fail to consolidate into dominant themes. Similarly, the single case of perfect prediction (precision = 1.000, recall = 1.000) should be interpreted with caution, as it reflects a stable thematic transition in the dataset rather than evidence of universal predictability. Finally, the temporal division by decade, although intuitive, may obscure finer year-by-year variations in thematic emergence and decline.

Despite these limitations, the proposed framework offers a scalable and generalizable template for the bibliometric analysis of thematic dynamics in communication-related disciplines and beyond. Its application to PSA research offers both retrospective clarity and forward-looking insight, serving scholars, educators, and clinicians seeking to navigate the evolving landscape of communication anxiety studies.
